# Differential Effects of *Rhodococcus equi* Virulence-Associated Proteins on Macrophages and Artificial Lipid Membranes

**DOI:** 10.1128/spectrum.03417-22

**Published:** 2023-02-14

**Authors:** Philipp Hansen, Thomas Haubenthal, Caroline Reiter, Jana Kniewel, Karla Bosse-Plois, Hartmut H. Niemann, Kristine von Bargen, Albert Haas

**Affiliations:** a Institute for Cell Biology, University of Bonn, Bonn, Germany; b Department of Chemistry, Bielefeld University, Bielefeld, Germany; LSU Health—New Orleans

**Keywords:** zoonosis, pneumonia, pathogen, pore forming, toxin

## Abstract

Virulence-associated protein A (VapA) of Rhodococcus equi is a pathogenicity factor required for the multiplication of virulent *R. equi* strains within spacious macrophage vacuoles. The production of VapA is characteristic for *R. equi* isolates from pneumonic foals. VapB and VapN proteins in *R. equi* isolates from infected pig (VapB) and cattle (VapN) have amino acid sequences very similar to VapA and consequently have been assumed to be its functional correlates. Using model membrane experiments, phagosome pH acidification analysis, lysosome size measurements, protein partitioning, and degradation assays, we provide support for the view that VapA and VapN promote intracellular multiplication of *R. equi* by neutralizing the pH of the *R. equi*-containing vacuole. VapB does not neutralize vacuole pH, is not as membrane active as VapA, and does not support intracellular multiplication. This study also shows that the size of the sometimes enormous *R. equi*-containing vacuoles or the partitioning of purified Vaps into organic phases are not features that have predictive value for virulence of *R. equi*, whereas the ability of Vaps to increase phagosome pH is coupled to virulence.

**IMPORTANCE** Rhodococcus equi is a major cause of life-threatening pneumonia in foals and occasionally in immunocompromised persons. Virulence-associated protein A (VapA) promotes *R. equi* multiplication in lung macrophages, which are the major host cells during foal infection. In this study, we compare cellular, biochemical, and biophysical phenotypes associated with VapA to those of VapB (typically produced by isolates from pigs) or VapN (isolates from cattle). Our data support the hypothesis that only some Vaps support multiplication in macrophages by pH neutralization of the phagosomes that *R. equi* inhabit.

## INTRODUCTION

Rhodococcus equi (occasionally named Rhodococcus hoagii or *Prescotella equi*) (1) is a Gram-positive soil actinomycete that is closely related to mycobacteria. It causes severe bronchopneumonia, particularly in young foals and, as a zoonotic disease, in immunocompromised humans such as AIDS patients (2, 3). The main host target of this intracellular pathogen are alveolar lung macrophages (4, 5), which track down and ingest inhaled microorganisms. Instead of being killed by macrophages, virulent *R. equi* multiplies in an unusual membrane-bound phagosome, the *R. equi*-containing vacuole (6–9). In addition to mammalian immune phagocytes, soil amoebas may provide an intracellular environment for *R. equi* (10). The key to intramacrophage multiplication is the abundant production of Virulence-associated protein A (VapA), whose gene is localized to virulence plasmids from foal isolates. VapA is an immunodominant surface protein; it neutralizes the pH of the endocytic and phagocytic continua of infected macrophages and allows the multiplication of *R. equi* within the vacuole, which can also contain lysosome material (8). The ability to neutralize its intracellular environment seems to be the key pathogenic feature toward macrophages (8). Temperatures above 33°C, as they occur inside potential hosts, serve as cues for VapA production, whereas lower environmental temperatures do not (11–13). A low pH promotes VapA production further, with high temperatures being the dominant regulator (14).

Whereas the *vapA*-containing plasmid (pVAPA) is almost invariably associated with isolates from diseased foals, *R. equi* isolated from pig lymph nodes typically carries a related plasmid, pVAPB, which contains the closest *vapA* orthologue, *vapB*. Isolates from cattle are predominantly associated with the pVAPN plasmid, from which the *vapA* orthologue *vapN* is expressed (15). Each bacterial isolate contains either only one plasmid type or none (15). A total of 21 *vap* genes and pseudogenes are known; four of these (*vapA*, *vapK1*, *vapK2*, and *vapN*) have demonstrated roles in macrophage infection, whereas the others are likely less or not at all relevant (16, 17). A recent intriguing study (7) showed that, against expectation, host specificity associated with the different pVAP types (pVAPA and foals, etc.) was not reproduced at the macrophage level. For example, some pVAPB-containing isolates from pigs also multiplied in experimentally infected equine macrophages (7). Therefore, the close association of certain plasmid types with certain hosts is not determined at the macrophage level.

VapB and VapN are highly homologous to VapA (78 and 59% amino acid sequence identities, respectively) and between each other (VapN shares 60% sequence identity with VapB) (16). Sequence identities are minimal in the Vap amino-terminal disordered regions and maximal in their carboxy-terminal portions, which form an 8-stranded β-barrel domain and a small α-helix (18–21). Deletion of *vapA* (22) or *vapN* (16) abrogates the multiplication of *R. equi* in murine macrophages, whereas deletion of the paralog *vapB* does not impact multiplication (17). This observation was surprising because, based on the very close structural relationship between VapA and VapB, their similar regulation, their immunodominance, and their presence in clinical isolates of *R. equi*, VapB was considered to be a functional homologue of VapA (11, 14, 18, 23–25).

To understand these differences in host cell modulation by proteins that seem so similar (18, 19, 21), we set out to study their effects on model membranes and on murine macrophage-like cell lines (henceforth referred to as “macrophages”), which have been widely used to study *R. equi* pathogenesis (6, 8, 26–29). We present results that indicate that the pH-raising effect in phagosomes of VapA and VapN, but not VapB, explains their virulence-supporting capacity.

## RESULTS

### VapA and VapN but not VapB increase phagosome pH and support intracellular multiplication.

**FIG 1:**
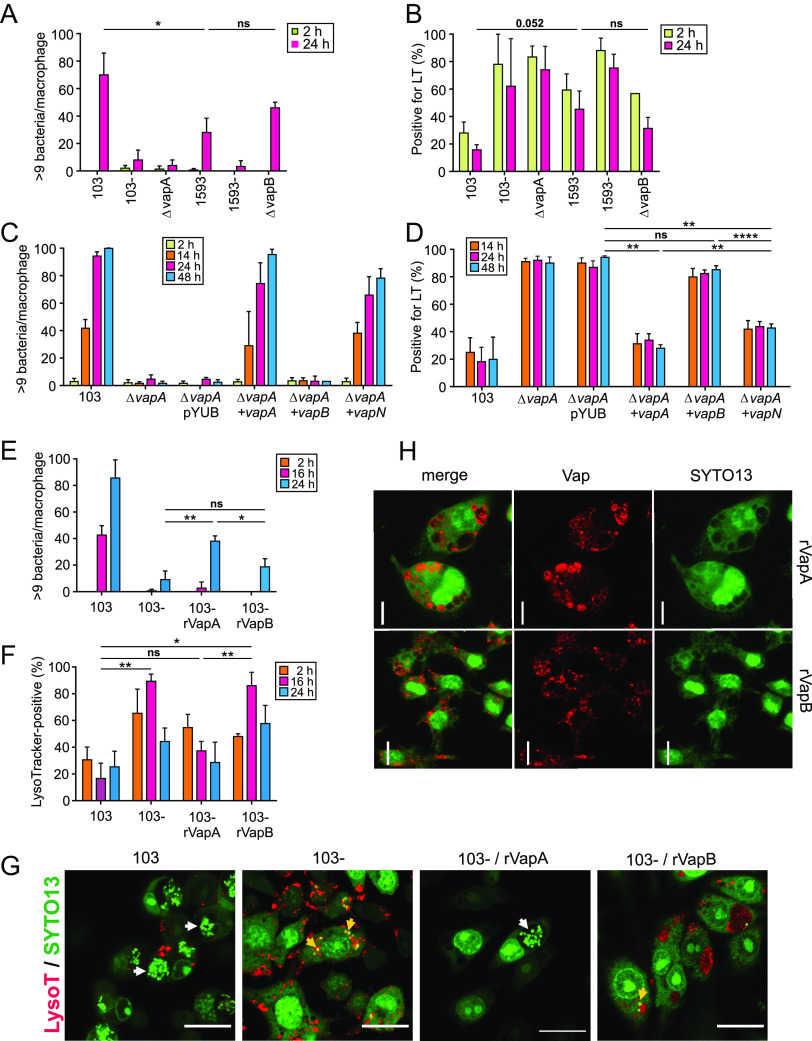
Intracellular multiplication and phagosome acidification in the presence of different Vaps. (A) *R. equi* strains carrying pVAPA (103) or not (103–) or pVAPB (PAM1593) or not (PAM1593–) or *vap* deletions (103Δ*vapA* and PAM1593Δ*vapB*) were used to infect RAW 264.7 macrophages, and infection was quantified at 2 h (before) and 24 h (after multiplication) by determining the proportion of infected macrophage with 10 or more bacteria from 50 macrophages per sample and experiment, as a proxy for robust multiplication (6, 8, 17). (B) Samples as in panel A were analyzed for colocalization of *R. equi* phagosomes with LT. (C) Strain 103Δ*vapA* was complemented with pYUB415 plasmids carrying either no insert (pYUB) or pYUB containing *vapA*, *vapB*, or *vapN*, all controlled by the *vapA* promoter and used to infect RAW 264.7 murine macrophages. After 2, 14, 24, or 48 h of infection, the samples were fixed, and infections were quantified microscopically as described above. (D) Samples as in panel C were analyzed for colocalization of *R. equi* phagosomes with LT. (E) Macrophages infected with avirulent strain 103– were extraneously supplied with either 10 μg/mL rVapA or rVapB, and multiplication was assessed as described above at the indicated times. (F) Samples as in panel E were analyzed for colocalization of *R. equi* phagosomes with LT. Statistical significance was calculated with the 16-h postinfection data because by 24 h most 103– bacteria have been killed, and those remaining seem to reside predominately in nonacidified phagosomes. (G) Micrographs from experiments as in panel F. White arrows point to multiplied bacteria in LT-negative phagosomes, and yellow arrows point to single bacteria in LT-positive compartments. Scale bars, 20 μm. Here and elsewhere, bacteria were visualized by using SYTO13, a fluorescent stain used particularly for DNA (bacteria and macrophage) but also for mRNA (explaining the host cell background stain). (H) The uptake of rVapA and rVapB was visualized by immunostaining after 24 h of addition. All data in panels A to F indicate the means and standard deviations (SD) from three independent experiments. Probability (*P* value) analyses here and below are marked as follows: n.s., not significant; the actual *P* value; or *, *P* < 0.05; **, *P* > 0.01; ***, *P* < 0.005; ****, *P* < 0.001. Scale bars, 20 μm.

VapB was assumed to be the functional homologue of VapA in strains with pVAPB (3, 14, 25). However, recent studies have shown that *vapB* knockout bacteria were not seriously affected in intramacrophage multiplication (17). Here, we have revisited the intracellular multiplication of the VapA-producing foal strain 103, of its isogenic virulence-plasmid-cured and VapA-less strain 103–, and of strain 103 lacking only *vapA*. As shown previously (8, 22, 30), loss of *vapA* sufficed to almost eliminate intramacrophage multiplication (Fig. 1A). Upon comparing the pVAPB-type isolate PAM1593 to the isogenic plasmid-less strain PAM1593– and to PAM1593 lacking the *vapB* gene, it became apparent that PAM1593 multiplied less efficiently than strain 103 and that the deletion of *vapB* did not at all decrease intracellular multiplication (Fig. 1A), supporting the notion that VapK1 and VapK2, but not VapB, are the true intracellular growth promoters for the porcine isolate (17).

Our previous work has indicated that raising the pH within the *R. equi* phagosome is the key factor that allows the bacteria to multiply intracellularly. Therefore, we tested whether the loss of VapB affects phagosome and lysosome acidification as much as does the presence of VapA (8). To this end, colocalization of phagosomes with the acidotropic dye LysoTracker Red (LT) was analyzed as a proxy for strongly acidified phagosomes and phagolysosomes (pH less than ~6.0). Phagosomes containing the pVAPB strain PAM1593 were more frequently than strain 103 associated with LT, indicating acidified compartments (Fig. 1B). Multiplication was reduced whenever the LT rates increased (Fig. 1A and B). These results indicated that VapB was not required for intracellular multiplication or for changes in phagosome pH.

Since VapB is not needed for intracellular multiplication of *R. equi* in its own genetic background, we tested whether the expression of either *vapA* or *vapB* would be able to functionally complement a *vapA* deletion strain in intracellular multiplication assays. We further included in our analysis the recently described *vapN* gene typical for bovine isolates (16). To this end, each of the three *vap* genes received the upstream expression-mediating sequences of *vapA* as transcriptional drivers to ensure equivalent expression. We observed that *vapA* functionally complemented the *vapA* mutation as it did *vapN* but not *vapB* (Fig. 1C). Successful complementation was paralleled by reduced LT colocalization (Fig. 1D). Apparently, VapA and VapN fulfilled similar functions, whereas VapB did not.

In a different approach, we added recombinant VapA (rVapA) to the growth media of infected macrophages to be taken up by endocytosis. rVapA complemented the absence of VapA in Δ*vapA* strains and in completely plasmidless strains (8, 31, 32). Addition of recombinant VapB (rVapB) to the infection media promoted multiplication of the plasmid-less strain 103– much less than did rVapA (Fig. 1E) (32). Again, reduced multiplication was correlated with an increased proportion of acidified phagosomes in the samples (Fig. 1F and G), further strengthening the link between phagosome acidification and growth arrest or killing. This lack in effect for VapB was not due to reduced VapB uptake (Fig. 1H).

### VapA causes spacious vacuole formation, whereas VapB and VapN do not.

**FIG 2:**
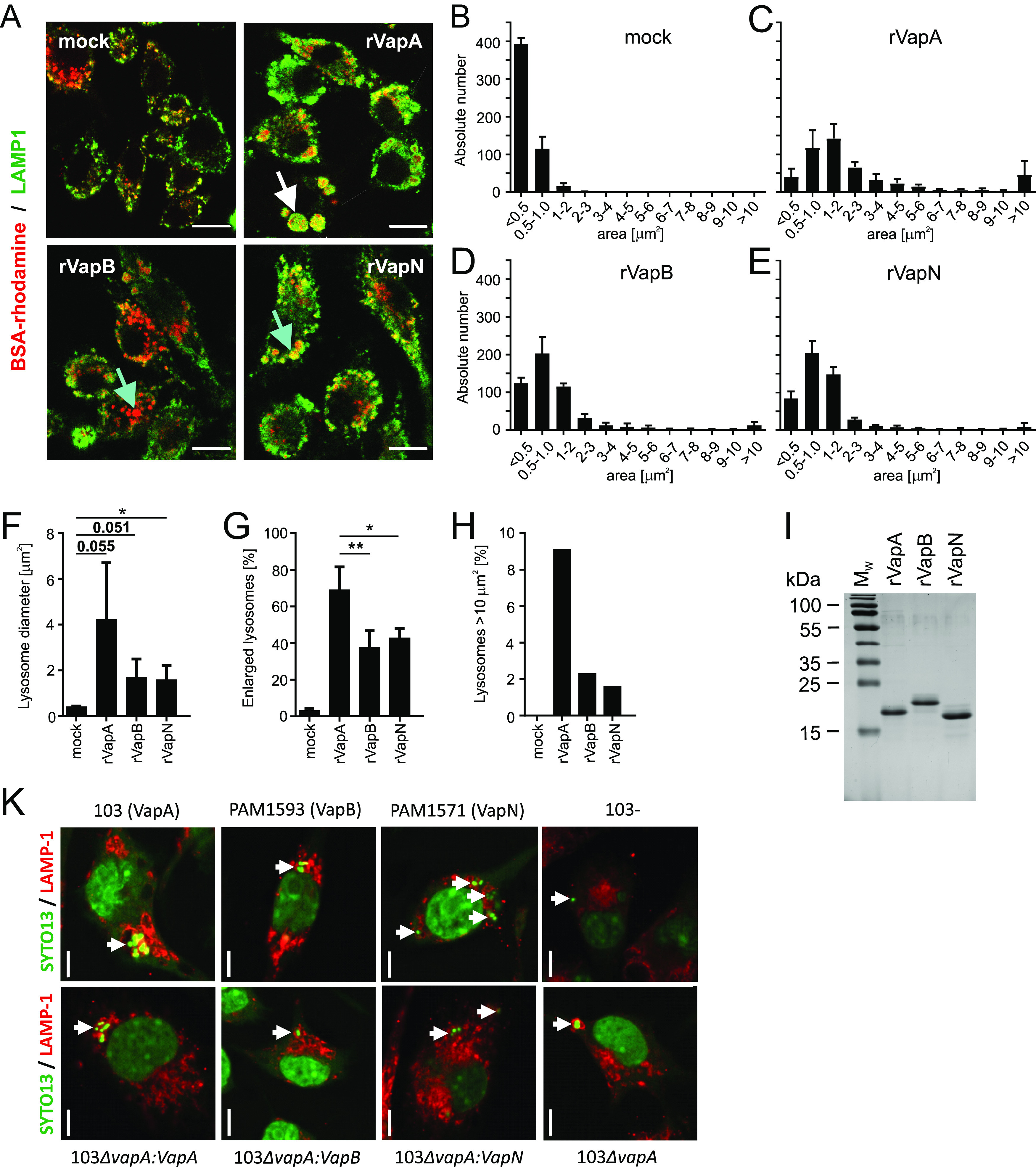
Vacuole formation in macrophages supplied with rVaps. (A) RAW 264.7 macrophages were incubated for 24 h in DMEM/FCS in the presence of 100 μg/mL rVapA, rVapB, or rVapN or none (mock). The white arrows denote a large vacuole; the blue arrows indicate little-altered lysosomes. (B to E) Quantification by micrograph analysis of lysosome areas in samples as in panel A, separated by area classes, yielding also the average lysosome diameters (F), the percentages of enlarged lysosomes (>1 μm^2^ in the focal plane) (G), and the proportions of lysosomes with an area of larger than 10 μm^2^ (H). (I) Colloidal Coomassie blue-stained polyacrylamide gel with a marker protein lane or 1 μg of rVap protein per lane. (K) Microphotographs of RAW 264.7 cells infected with the indicated *R. equi* strains for 24 h, followed by visualization of bacteria and nuclei with SYTO13 and counterstained with LAMP-1 antibody. Arrows point to phagosomes. Data in panels B to G show the means and SD and H the means from three independent experiments with 50 macrophages analyzed per sample and experiment. Statistical analysis was performed as described for Fig. 1. Scale bars, 10 μm (A) and 5 μm (K).

Virulent *R. equi* reside in a spacious phagosome derivative, the *Rhodococcus*-containing vacuole which may eventually fill much of the macrophage cytoplasm (8, 9, 27). We have observed that the generation of such a big compartment with numerous luminal membrane vesicles can be experimentally mimicked by the addition of rVapA to the culture media in the absence of the bacteria (as described previously [8] and as shown in Fig. 2A and C). Here, we tested whether rVapB or rVapN (Fig. 2A to H) would also be able to cause vacuole formation in macrophage-like cells. To visualize vacuoles, macrophages were cultivated in the presence of rhodamine-labeled bovine serum albumin (BSA) overnight to label lysosomes (8); then, rVaps were added to the culture media for 24 h, the macrophages were fixed, and lysosome-associated membrane protein-1 (LAMP-1) was visualized by immunofluorescence microscopy. In this way, LAMP-1 and BSA-rhodamine served as membrane and luminal lysosome markers, respectively. As a peculiarity, LAMP-1 also stains internal vesicles of rVapA-induced vacuoles (8). Very large vacuoles can be clearly seen in rVapA-treated samples but rarely in VapB- or VapN-treated samples (Fig. 2G and H). We calculated the areas of LAMP-1-positive structures in the focal plane and plotted them against their frequencies. All Vap proteins led to larger vacuoles compared to the mock-treated sample (Fig. 2B to H). This shift in size was most pronounced with rVapA-treated samples (Fig. 2C), where most lysosomes were enlarged (>1 μm^2^; Fig. 2G), often having an apparent area larger than 10 μm^2^ (Fig. 2H). VapN did not enlarge lysosomes more than VapB did (Fig. 2F and G), yet only VapN potently supported *R. equi* multiplication (Fig. 1C), indicating that generation of large vacuolated lysosomes (Fig. 2H) by the action of Vap proteins is not required for virulence.

These data from “sterile infections” (Vaps only) agree well with data from infected macrophages (Fig. 2K): VapA-producing strain 103 multiplies and localizes to often one, sometimes two large or very large vacuoles per macrophage, and so does the *vapA*-complemented strain 103Δ*vapA*; the VapB-producing strain PAM1593 multiplies little and hence typically stays in a tight phagosome; the VapN-producing isolate PAM1571 multiplies but typically resides in several, usually two to four, separated small vacuoles (the precise determination of where one vacuole ends and the other begins is often difficult, particularly since potential vacuole perimeter markers, such as LAMP-1, are found not only in the vacuole membrane but also within the vacuole); and the Vap-complemented Δ*vapA* strains deliver the corresponding data and further indicate that the vacuole size is largely determined by the expressed Vap type and not the bacterial chromosome.

### VapA inhibits acidification and intraphagosomal proteolysis strongly, whereas VapN inhibits acidification and intraphagosomal proteolysis partially and VapB barely.

**FIG 3:**
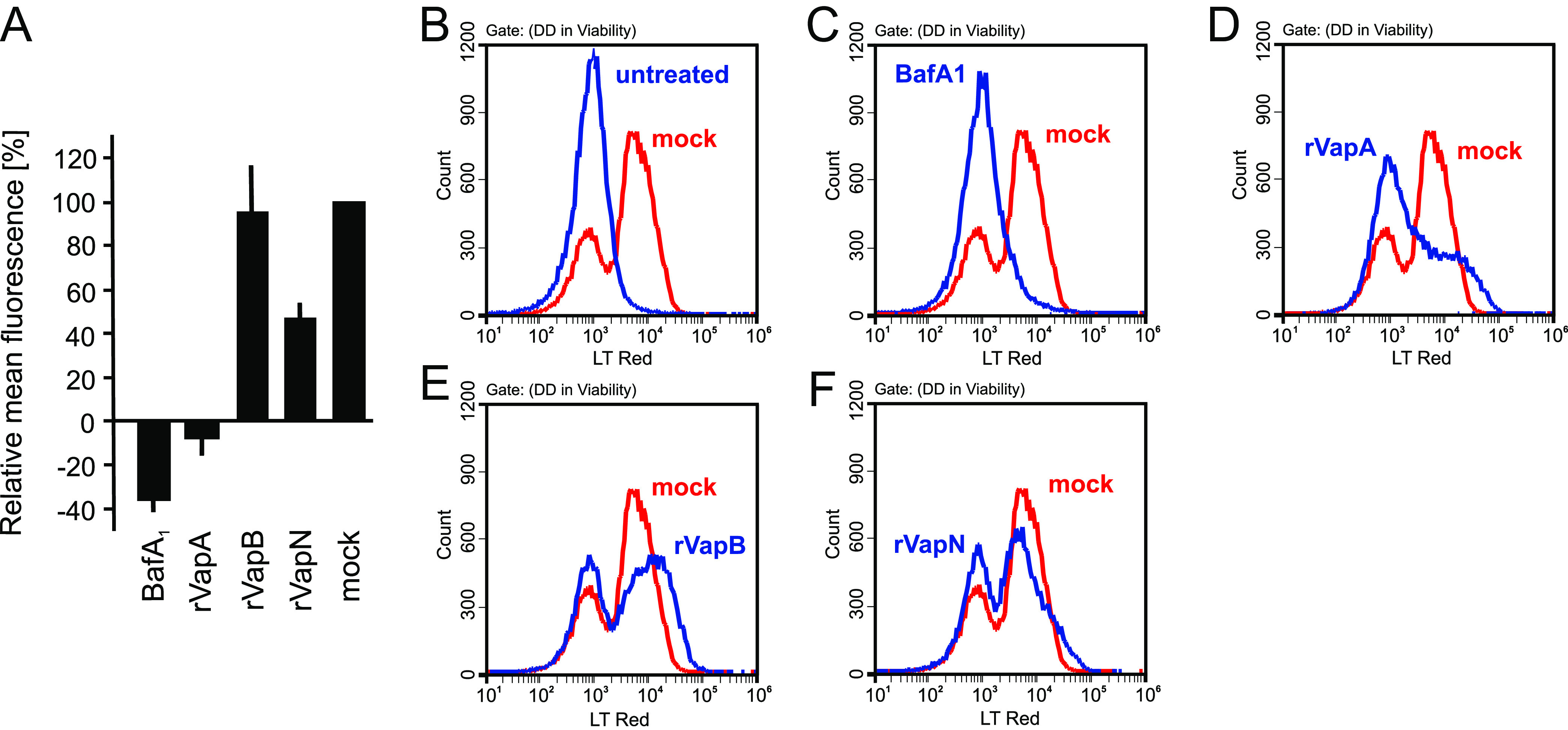
Flow cytometry analysis of acidification in Vap-treated macrophages. RAW 264.7 macrophages were incubated with either 50 μg/mL rVapA, rVapB, or rVapN or 40 nM bafilomycin A_1_ or without additives (mock) for 23.5 h at 37°C, followed by incubation with 200 nM LT for 30 min and immediate analysis for LysoTracker content by flow cytometry. (A) Mean fluorescence and SD of cells from three independent experiments, with 100% corresponding to the average intensities of mock samples as displayed in panels B to F and 0% corresponding to the average intensity of untreated samples as in panel B. (B to F) LT intensity distributions of cell populations from one representative experiment. The red lines in all graphs represents cells with LT only added (mock). The blue lines represent no LT added (B), BafA_1_ added (C), rVapA added (D), (E) rVapB added (E), or rVapN added (F).

Previous work has shown that addition of rVapA to macrophages reduces lysosome acidification and staining for LT, i.e., lysosomes have a near-neutral pH (8). To test whether this was also true for VapB and VapN, we added rVapA, rVapB, or rVapN to macrophages, or we added bafilomycin A_1_ (BafA_1_), which is a potent inhibitor of the lysosome-acidifying ATPase (33), and quantified LT accumulation in the absence of an infection. Macrophages that were mock treated showed a strong fluorescence peak at around 8,000 fluorescent units (Fig. 3B, red), whereas LT-less cells peaked at around 900 U (Fig. 3B, blue), just as BafA_1_-treated cells did in the presence of LT (Fig. 3C). Treatment with rVapA removed clearly more of the LT “shoulder” than did rVapN; many cells even shifted to the left into the “completely nonstained” area (Fig. 3D). In samples with rVapB addition, LT fluorescence was in fact higher than in the “LT only” control (Fig. 3C versus Fig. 3F). The rVapN samples show the same percentage of acidified cells as rVapB cells, yet the right peak was shifted to the left, indicating reduced acidification (Fig. 3C versus Fig. 3F). The analysis of the mean fluorescence of cells in the different samples revealed similar trends, i.e., rVapA action reduced LT staining most, rVapN intermediately, and rVapB least (Fig. 3A). Therefore, at least in in murine macrophages, rVapA abrogates robust lysosomal acidification in whole cells completely, rVapB abrogates robust lysosomal acidification in some cells partially, and rVapN reduced LT accumulation in most cells but rarely abrogated it. These data complement the data presented above for macrophages containing the *vapB*-complemented Δ*vapA* bacteria, where the expression of *vapB* did only very little reduce phagosome colocalization with LT, yet *vapN* did almost as much as *vapA* (Fig. 1D).

**FIG 4:**
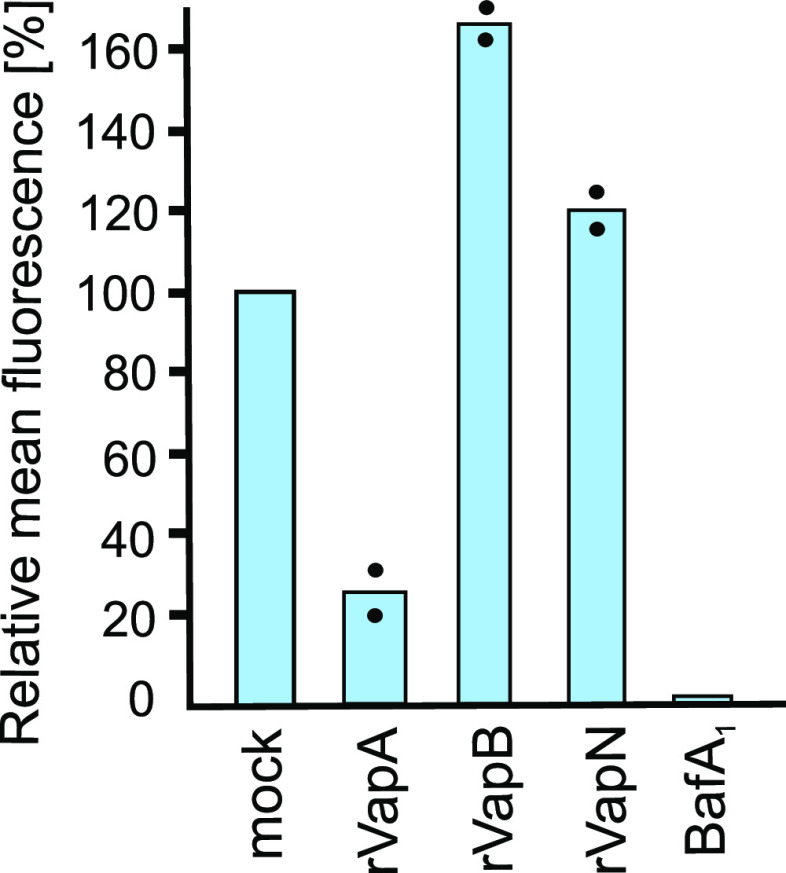
Lysosomal proteolysis in Vap-treated macrophages. RAW 264.7 macrophages were incubated for 20 h in medium containing 50 μg/mL of a particular rVap protein, no protein (mock), or bafilomycin A_1_ (BafA_1_). Then, 10 μg/mL DQ-BSA in medium was added for 2 h (uptake and digestion), and the medium was exchanged for fresh medium without DQ-BSA, followed by incubation for another 2 h (more digestion). Cells were harvested and analyzed for DQ-BSA degradation by flow cytometry. The mean fluorescence of cells in the BafA_1_ control was set as 0% digestion and in the mock control sample as 100%. Data points are from two independent experiments.

One major biological role of lysosome acidification is the activation of lysosome hydrolases selectively in the lysosome (34) and the faster degradation of proteins. Here, we have tested whether the reduction in LT colocalization by VapA also resulted in reduced hydrolase (protease) activity. To this end, we used a flow cytometry-based proteolysis assay with a BSA that had been densely labeled with fluorophore such that its fluorescence is self-quenched. Once this DQ-BSA is digested, the resulting peptides are released and fluorescence increases (hence, “DQ-BSA” = “dequenched BSA”). Here, rVapA reduced intralysosomal DQ-BSA digestion almost as potently as did inhibiting lysosome acidification (BafA_1_), whereas treatment of macrophages with rVapB or rVapN did not decrease digestion (Fig. 4). These data reflect the strong effect VapA has on lysosome acidification. The increased dequenching signal in cells treated with rVapB (Fig. 4) could well be a consequence of the population of macrophages that stain actually stronger for LT (i.e., they have more acidic compartments) than mock cells (Fig. 3F, the blue line reaches right of red line). In these cells, increased acidification may further stimulate proteolysis (35) and, at the same time, the somewhat enlarged lysosomes (Fig. 2G) may provide more space for the dequenching reaction after BSA digestion. rVapN would cause a similarly increased lysosome size (Fig. 1F), but a lack of increased pH would mean no boost of proteolysis (Fig. 3). In any instance, the result that VapN-containing macrophages show robust DQ-BSA digestion makes us conclude that proteolytic potency is not a decisive factor for intracellular multiplication or host restriction of *R. equi*.

### Binding to and permeabilization of liposomes by VapA, VapB, and VapN.

VapA collapses intracellular proton gradients by membrane permeabilization rather than by inhibiting the activity of the lysosomal proton-pumping ATPase (8). We have previously shown that recombinant VapA permeabilizes liposomes composed of purified bovine brain total lipids sufficiently to release 5(6)-carboxyfluorescein (CF; molecular weight, 376.3 g/mol) (8). In addition, it has been reported that VapA binds to liposomes composed of phosphatidylcholine and phosphatidic acid (80:20) at an acidic pH but not to liposomes composed of phosphatidylcholine only and not at a pH of 7.4 (32). We extended these studies by studying rVapB for its capabilities to bind to liposome membranes and by testing different complex lipid mixtures, i.e., asolectin (lipid preparation from soybean), total brain lipids from pig, or a “lysosome lipid” mix based on a mouse lysosome lipid composition published previously (36), for their Vap affinity.

**FIG 5:**
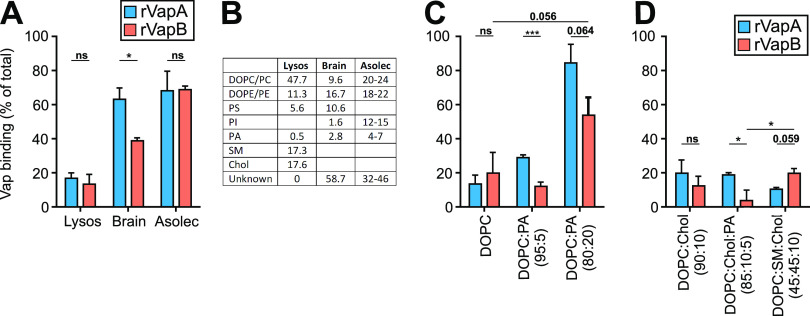
VapA/B binding to liposomes. (A) 200-nm-diameter liposomes were generated by extrusion of mixtures of lipids as indicated in panel B using a “lysosome-mimicking composition” (Lysos), a total lipid extract from pig brain (Brain), and a lipid mixture from soybean, asolectin (Asolec). Compositions are expressed as in mol% for Lyso and % (wt/wt) for brain and asolec lipids. Five μg of rVapA or rVapB was added for 1 h at 37°C, and the liposomes were examined at pH 4.5. Attached proteins were reisolated by floatation, followed by densitometric quantification from colloidal Coomassie blue-stained polyacrylamide gels. (C and D) Same as in panel A but using liposomes made from different lipid compositions. Data represent the means and SD from three independent experiments. Statistical analysis was performed as described in Fig. 1. Chol, cholesterol; DOPC, 1,2-dioleoyl-*sn*-glycero-3-phosphocholine; DOPE, 1,2-dioleoyl-*sn*-glycero-3-phosphoethanolamine; PS, phosphatidylserine; PI, phosphatidylinositol; SM, sphingomyelin; PA, phosphatidic acid.

We were surprised to observe that VapA binding to lipids was minimal with the lysosome membrane mimic (Fig. 5A), although lysosomes were expected to contain the biologically most relevant lipid composition. As seen previously for VapA (32), phosphatidic acid (PA) stimulated VapB binding to lysosomes (Fig. 5C), although Wright et al. (32) have reported a lack of VapB binding to liposomes under similar conditions. One explanation for this discrepancy might be that in Wright et al. (32) the binding signal of rVapA was low and that therefore an even weaker VapB binding signal might not have been detectable. In addition, these authors used 1-μm-diameter liposomes, whereas we used 0.2-μm liposomes, and Vaps might prefer one membrane curvature over another just like BAR proteins do. BAR proteins are a family of crescent-shaped protein dimers that bind to and support a specific membrane curvature (37). Since VapA is a membrane-active protein, it might well bind more strongly to one membrane curvature over another, but no comparative binding studies have yet been published. Cholesterol, which is required for the binding and function of many bacterial membrane-active proteins (38), influenced the binding of neither rVapA nor rVapB except that rVapA bound better when PA was also present (Fig. 5D). However, binding was about as low as to liposomes containing 1,2-dioleoyl-*sn*-glycero-3-phosphocholine (DOPC) alone (Fig. 5C). In summary, the binding of rVapA to model liposomes at acidic pH was more pronounced than the binding of rVapB when PA was present and in brain lipid liposomes.

**FIG 6:**
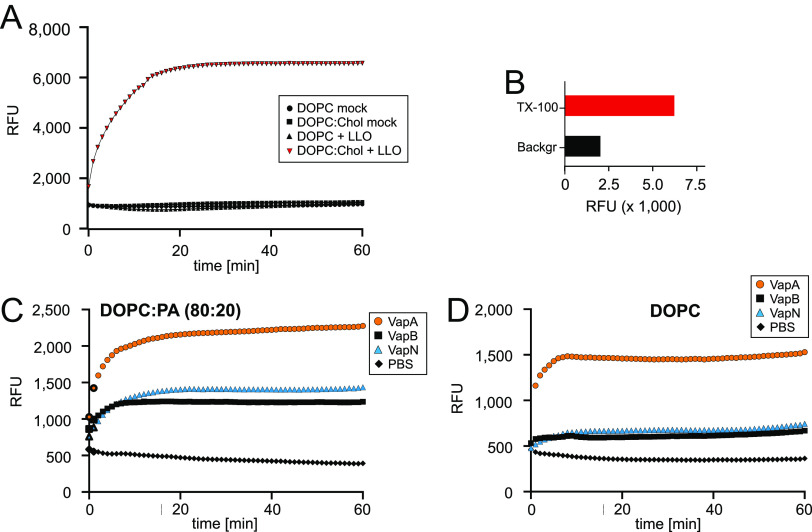
Permeabilization of liposomes by rVaps. Liposomes (200-nm diameter) with enclosed self-quenching CF were generated by lipid extrusion using DOPC:Chol (80:20) (A and B), DOPC:PA (80:20) (C), or DOPC only (D). CF dequenching as a measure of release was recorded over a 60-min period. (A) Listeriolysin O (50 μg/mL) was added at 0 min. (B) Fluorescence signals in panel A at the start of the experiment (Backgr) and after maximum dequenching by addition of TX-100 (TX-100). (C and D) rVapA, rVapB, or rVapN (50 μg/mL) was added at 0 min, and CF release was recorded over 60 min. PBS, buffer only. RFU, relative fluorescence units. Data in panels A and B show means from two independent experiments; data in panels C and D show means from six independent experiments, with error bars omitted for clarity.

In addition to membrane binding, we also analyzed the capability of rVaps to permeabilize liposome membranes for CF. Leakage was quantified using liposomes made from either DOPC alone or from DOPC mixed with PA in an 80:20 molar ratio. The liposomes were prepared in the presence of CF at a high concentration, which leads to self-quenching, and nonincorporated CF was removed by gel filtration. When these liposomes leak CF, the self-quenching is terminated, and CF emits full fluorescence, a feature that is maximized by the addition of the membrane-disrupting detergent Triton X-100 (TX-100; Fig. 6B). We first quality-checked our liposome permeabilization assay by using the well-investigated bacterial membrane disruptor listeriolysin O (LLO) from the food-pathogen Listeria monocytogenes. LLO requires membrane cholesterol to form large pores in biomembranes for CF to leak (39), as seen in the present study (Fig. 6A).

As for *R. equi* proteins, rVapA clearly was the most potent membrane disruptor when membranes were composed of 80% DOPC and 20% PA, but VapB and VapN also generated some permeability changes (Fig. 6C), and the same was true for DOPC-only liposomes (Fig. 6D). The almost straight “PBS” trail (Fig. 6C and D) demonstrated liposome integrity during the time frame of the experiment. The fact that fluorescence in the mock-treated sample decreased somewhat over time was not the consequence of membrane leakage because this would have increased the fluorescence rather than decreased it. The modest decrease is, rather, the consequence of fluorescence bleaching of CF by the many fluorescence detection cycles over time. The addition of either rVapB or rVapN to liposomes caused only little disturbance (only a few percent of the maximum signal). The addition of rVapA resulted in a ca. 15% of maximum increase in fluorescence signal (Fig. 6D and data not shown). The fluorescence intensities increased considerably with either rVap protein when DOPC:PA (80:20) liposomes were used (Fig. 6C), to which VapA and VapB bind particularly well. Remarkably, the onset of the permeabilization and therefore the action of Vaps happened within only a few minutes.

These data indicated that of the three Vaps, VapA had the strongest potential for causing CF release and that VapB and VapN have a similar reduced potential in this setup. This result raises the question of why VapN supported intracellular multiplication of *R. equi*, whereas VapB did not. We argue that one of the reasons is that a local permeabilization of biomembranes big enough to allow CF release is an exaggerated activity of VapA that occurs rarely *in vivo*, where the pathologically relevant activity is the release of much smaller protons or hydronium (protonated water). It is possible that the very low rupturing activity in phagosomes is due to the high density of glycosylated proteins in phagosome and lysosome membranes or membrane repair mechanisms. Having seen many transmission electron microscope photos of *R. equi*-infected cells, we have never seen a ruptured phagosome unless the infected macrophage was in the process of necrosis, leading macrophage death and release of multiplied bacteria (40). Along the same line, a recent study on *R. equi*-containing mouse macrophages showed that only a small portion of phagosomes was positive for galectin-3, a probe for damaged endocytic membranes, and these infrequent damages occurred independent of VapA production (26). Therefore, although the CF release phenomenon may be exaggerated or play a role only very late in infection, it is an excellent measure of how membrane active a Vap is. We propose that VapA is a generally strong membrane-active compound, that VapN a weaker one, and that VapB is still weaker, with the exact permeabilization activities being dependent on the precise membrane composition. VapN would generally be less potent than VapA in membrane disordering, as reflected in experimental CF release but, on the other hand, it is strong enough to reduce phagosome and lysosome acidification (Fig. 1D and Fig. 3D), demonstrating its ability to induce permeability increases *in vivo*. VapB binds more weakly than VapA to our minimalist model membranes if they contain PA (Fig. 5C and D), and VapB is weakly membrane disturbing (Fig. 6D) and therefore cannot collapse pH gradients across the phagosome membrane (Fig. 1D). This seems to be the clue to answering the question why VapB cannot functionally replace VapA in virulence.

### All Vap proteins partition into the Triton X-114 organic phase.

**FIG 7:**
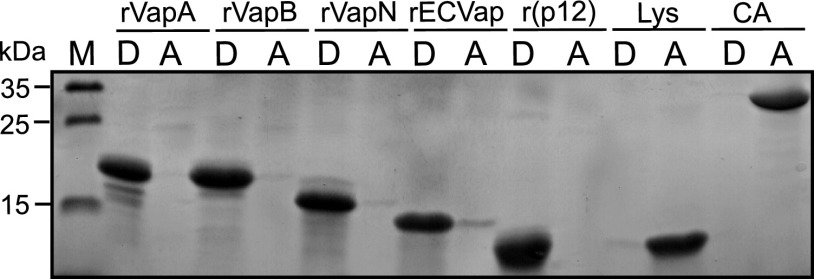
Affinity of recombinant Vaps for TX-114. Each indicated protein was solubilized in 2% Triton X-114/TBS solution and phase separation was performed into aqueous (lanes A) and detergent (lanes D) TX-114 phases. Equal sample sizes of each protein solution were applied to a 12% polyacrylamide gel, which was stained for protein by colloidal Coomassie blue. CA, carbonic anhydrase; Lys, lysozyme; M, molecular weight marker. The gel is representative for three independent experiments.

VapA readily partitions from aqueous phase into the Triton X-114 organic hydrophobic phase (TX-114; not to be confused with TX-100) (41). VapB also, surprisingly, partitions into the organic phase (42). This observation was originally ascribed to the palmitoylation of VapA (42), yet VapA does not possess a cysteine residue that could serve as a palmitate acceptor and is overall quite hydrophilic when modeled from crystal structures of other Vap proteins (18–20). Also, mass spectrometry analysis in our laboratory did not yield the molecular masses expected for modified VapA, regardless of whether VapA was purified from *R. equi* or from recombinant Escherichia coli (not shown). It is therefore enigmatic why VapA partitions into TX-114, but we reasoned that this unusual feature could be a readout for the virulence-supporting and membrane-disrupting properties of VapA. However, all of the purified recombinant Vaps tested here—rVapA, rVapB, rVapN, and rECVap (the VapA homologue from an apathogenic Escherichia coli strain), as well as the recombinant protease-resistant and membrane-active 12 kDa-fragment of VapA (p12)—distributed mostly (rECVap) or completely (rVapA) into the organic phase. The cytosolic negative-control proteins carbonic anhydrase and lysozyme distributed exclusively into the aqueous phase (Fig. 7). This experiment demonstrated that TX-114 partitioning is, surprisingly, a general feature of all tested Vap proteins, including VapB and ECVap. This result is hard to understand because the Vaps and p12 have what is likely a stable barrel structure which would not be expected to change dramatically upon contact with TX-114 and expose hydrophobic portions. In any instance, we show here that this unusual feature cannot be used as a criterion to quickly assess Vap activities for membranes and toward phagocytic cells.

### None of several point mutations approximating the VapB sequence to VapA increases virulence-related activities.

**FIG 8:**
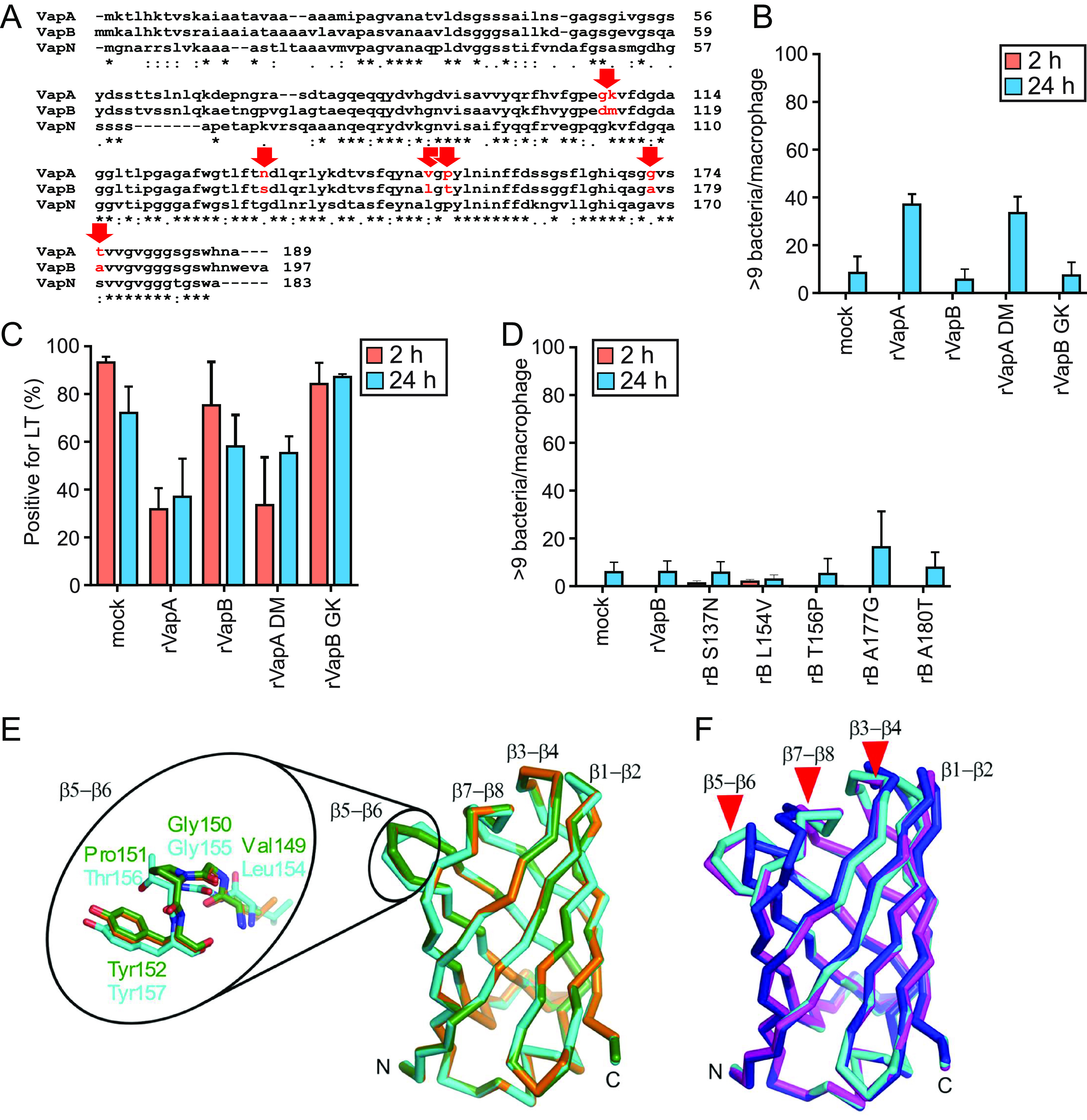
Test in macrophages of Vaps carrying amino acid exchanges. (A) Amino acid sequence comparison of VapA and VapB. Stars indicate identity between VapA and VapB, semicolons indicate amino acids with very similar characteristics, and dots with somewhat similar characteristics. Secreted mature VapA commences with amino acid residue 31, the protease-resistant portion of VapA with residue 80. Positions of amino acid exchanges are marked by red arrows. Accession numbers: VapA, GenBank MBP0079885.1; VapB, NCBI Reference Sequence WP_012532617.1. (B) Bacterial multiplication in macrophages supplied with 100 μg/mL rVaps. (C) Quantitation of LT colocalization with phagosomes. Analyses were performed as described for Fig. 1. (D) Macrophages received 100 μg/mL modified rVapB (rB) during and after infection. All quantitative data represent the means and SD from three independent experiments. (E) Overlay of AlphaFold2 structure predictions for VapA (green, AF-Q9EUL9-F1-model_v4), VapB (cyan, AF-B4F366-F1-model_v4), and VapN (orange, A0A0F6WFP7-F1-model_v4). The loop between strands β5 and β6 (residues 149 to 152 in VapA and residues 154 to 157 in VapB) is shown as a stick model enlarged on the left. (F) Overlay of the VapB structure predicted by AlphaFold2 (cyan) with structures experimentally determined by X-ray crystallography: PDB ID 4cv7 (magenta) and PDB ID 7b1z, chain A (blue). The largest structural deviations are marked by red arrowheads.

The amino acid sequence differences between the conserved carboxy-terminal portion of VapA and VapB are few and mostly conservative in nature (Fig. 8A). Therefore, we tested whether the change in VapB of single amino acid residues that differ between VapB and VapA would be able to change VapB features toward VapA. We concentrated on the p12 portion of VapA, which is sufficient to promote intracellular growth of *R. equi* (not shown) and which starts at Gly80. We introduced one each of the following amino acid changes: S137N, L154V, T156P, A177G, and A180T (Fig. 8D) (the numbers are as in the full-length protein containing the secretion sequence which is omitted from the recombinant proteins produced in E. coli). None of these single changes altered VapB’s ability to support intracellular multiplication significantly when added as modified rVapB to macrophages infected with strain 103– (Fig. 8D). Also, LysoTracker staining of their phagosomes was unaltered (not shown).

We further tested mutations in the only Vap positions where two adjacent amino acids differ between VapA and VapB. In VapB, amino acids D112 and M113 were changed to G112/K113 (VapA). Conversely, in VapA G107 and K108 were changed to D107/M108 (VapB). Again, VapB was not “activated” by the charge-changing mutations and did not support intracellular growth. Conversely, VapA did not lose any of its growth-promoting activity by removing the positive charge from K108 plus introducing the G107 negative charge (Fig. 8B). As predicted from the data presented above, neither of these double mutants effected the LT colocalization with 103– when added to macrophages as recombinant proteins (Fig. 8C).

To date, experimental structures are available for VapB but not VapA or VapN. Therefore, we used the arguably best structures available, namely, those predicted by the AlphaFold2 artificial intelligence structure prediction program. Overlay of these structures shows that the backbone traces of VapA, VapB, and VapN are virtually identical (Fig. 8E). Only the backbone of the VapB model shows slight deviations in the short loop connecting strands β5 and β6. This difference is due to the presence of Thr156 in VapB as opposed to a proline in VapA and VapN at residues 151 and 147, respectively (Fig. 8E, stick model). However, we show here that the T156P mutation had no effect on the activity of rVapB (Fig. 8D). Therefore, this structural difference between VapB versus VapA and VapN does not seem to be functionally important. In fact, the differences between the two experimental (20, 21) and the AlphaFold2 predicted structures of VapB are bigger than the differences between the AlphaFold2 predictions for all three different Vaps, particularly in the loop regions at the top of the molecule (Fig. 8F, arrowheads). In summary, structure comparison yielded no further clues to explain the different activities of these Vaps. Although much or all of the VapA membrane activities are localized to the conserved structure (8) shown in Fig. 8E, some of the observed activity differences might be caused by different properties of the unstructured amino-terminal portions of the Vaps.

## DISCUSSION

We have shown that VapA, VapB, and VapN affect membrane properties differently, which explains at least some of their intracellular activities or the lack thereof. The hypothesis that the major pathogenetically relevant activity of VapA is to collapse the proton gradient across phagosome and/or lysosome membranes was supported by all results obtained here: VapA pH neutralized phagosomes in macrophages, and VapB did not; VapA inhibited protein degradation in lysosomes, and VapB did not; VapA bound and particularly permeabilized liposome membranes strongly, and VapB did so more weakly; and VapA supported intracellular growth of avirulent *R. equi* regardless of whether they produced the protein themselves or whether it was provided in purified form, and VapB did not. These data strongly imply that the inability of VapB to support *R. equi* growth is eventually due to its failure to permeabilize membranes for protons. VapN is intermediary in the various activities yet stands closer to VapA than to VapB. Most importantly, rVapN collapsed lysosome proton gradients in living cells almost as efficiently as did VapA, as evidenced by the LT colocalization experiments (Fig. 1D). In summary, we hypothesize that although VapA seems clearly more “membrane active” than VapN, VapN is active enough to fulfill its biological role. A complete collapse of the proton gradient is likely not necessary for bacterial growth, and raising the pH from 5.1 (8) to ~6.2 (where phagosomes would turn LT negative) might suffice to support intracellular growth (8). On the other hand, a pH of 6.2 would promote intracellular protein digestion considerably over a pH of 7.2 (43), as seen in the rVapN sample (Fig. 4). Interestingly, full proteolytic capacity in phagosomes affects *R. equi* multiplication little or not at all. Finally, we provide evidence that lysosome vacuolization by Vap proteins is not a useful readout to assess virulence features of *R. equi*. This is different from other vacuolizing agents such as the structurally unrelated secreted VacA protein from Helicobacter pylori, which vacuolizes late endosomes and which is secreted almost exclusively by virulent strains (44). Furthermore, the unusual partitioning behavior of Vaps into the organic TX-114 phase does not reflect virulence-related potential of the respective Vap either. The fact that single amino acid changes in the conserved region of VapB did not activate a membrane-permeabilizing activity may point to the need to change several amino acids to turn VapB into a protein with VapA activities.

It is apparent from our data and those from others (32) that PA strongly enhances VapA action on biomembrane mimics. Whether PA really is a central binding partner of VapA during an infection remains to be shown. PA is a rare lipid amounting to approximately 0.2% of all lipids in mouse cell lysosomes (36), yet 5% PA in liposomes is not enough to clearly stimulate Vap binding (Fig. 5C) (32). Although it is possible that generation of PA by phospholipase D might yield increased local concentrations which could then be recognized by VapA, it is similarly possible that hitherto-unidentified acidic phospholipids are preferred targets *in vivo*. For example, the acidic phosphatidylserine, which contributes almost 10% of the phospholipids in latex bead-containing phagolysosomes (45), may play a role.

Preliminary work has indicated that VapA may be membrane active by increasing membrane rigidity in a way quite different from known pore-forming proteins (46). This observation may point biophysical researchers the direction where to look to define why, at the structural level, VapA quite efficiently permeabilizes membranes for protons and VapB does not.

## MATERIALS AND METHODS

### Cell culture and bacteria.

RAW 264.7 macrophage-like cells (47) were grown at 37°C and 5% CO_2_ in Dulbecco modified Eagle medium (DMEM) high glucose (Gibco/Life Technologies, catalog no. 31053028)/10% fetal calf serum (FCS; Sigma-Aldrich F7524, lot 035M3394)/1% (vol/vol) GlutaMAX (Gibco/Fisher Scientific, catalog no. 35050061). The bacterial strains used in this study are listed in Table 1. *R. equi* was cultivated in brain heart infusion broth (BHI; Becton Dickinson) at 30°C unless indicated otherwise, and Escherichia coli was cultivated in Luria broth (Carl Roth [Karlsruhe, Germany], catalog no. X964.1) at the indicated temperatures.

**TABLE 1:** Bacterial strains used in this study

Strain	Description	Source	Source or reference
Escherichia coli NEB5α F^1^I^q^	Cloning strain.	New England Biolabs (catalog no. C2987)	
Escherichia coli BL21(DE3)pLysS	Strain for recombinant protein expression.	New England Biolabs (catalog no. C3010)	
*R. equi* 103+	Isolated from a foal with pneumonia. Possesses pVAPA	J. F. Prescott (University of Guelph, Guelph, Canada)	58
*R. equi* 103–	Plasmid-cured derivative of 103+	Generated by repeated passage at 39°C	30
*R. equi* 103+*ΔvapA*	103+ with a replacement of *vapA* by *aacC4* apramycin resistance gene	This laboratory	30
*R. equi* PAM1593+	Isolated from an AIDS patient. Possesses pVAPB	M. Gobernado (University Clinic La Fe, Valencia, Spain)	15
*R. equi* PAM1593–	Plasmid-cured derivative of PAM1593+	Generated by repeated passage at 39°C	This study
*R. equi* PAM1593+*ΔvapB*	PAM1593+ with *vapB* replaced by *aacC4* apramycin resistance gene	This laboratory	This study
*R. equi* PAM1571+	Isolated from a foal mediastinal lymph node; possesses pVAPN	J. A. Vázquez-Boland (University of Edinburgh, Edinburgh, UK)	16

### Gene cloning.

Table 2 contains an overview of the plasmids used in this study. Recombinant E. coli Vap (rECVap) was expressed from the hexahistidine tag vector pET28a(+) (Novagen, catalog no. 69864-3). The synthetic gene (Life Technologies/Thermo Fisher Scientific; construct 11AA7F4P; see Fig. S1 in the supplemental material) was cut with SacI and NdeI and ligated into the vector cut with the same enzymes using T4 ligase.

**TABLE 2:** Plasmids used in this study

Plasmid	Description	Selectable marker	Source	Source or reference
pET28a(+)	N-terminal His_6_ tag vector for expression in E. coli	KanR	Novagen, catalog no. 69864-3	
pETite-N-His	N-terminal His_6_ tag vector for expression in E. coli	KanR	Lucigen, catalog no. 49001-1	
pET28a(+) N-His-ECVap	Plasmid for the expression of N-terminal tagged recombinant VapA orthologue from E. coli	KanR		This study
pETite-N-His-VapB	Plasmid for the expression of N-terminal His_6_-tagged VapB with signal sequence; *vapB* was amplified from pVAPN1593	KanR		This study
pETite-N-His-VapB SS Deletion	pETite-N-His_6_-VapB without N-terminal secretion signal sequence	KanR		This study
pETite-N-His-VapN	Plasmid for the expression of N-terminal His_6_-tagged VapN without signal sequence in E. coli; *vapN* was amplified from pVAPN1571	KanR		This study
pET28a(+)-N-His-VapA	Plasmid for the expression of N-terminal His_6_-tagged VapA in E. coli	KanR		8
pET28a(+)-N-His-VapA-DM	pET28a(+)VapA with two point mutations (G112D and K113M)	KanR		This study
pETite-N-His-VapB mutants	pETite-N-His-VapB without secretion signal sequence and a single point mutation each: A177G, A180T, L154V, S137N, or T156P	KanR		This study
pYUB415	E. coli-Mycobacterium shuttle vector	HygR	W. Jacobs (Albert Einstein College, Bronx, NY)	58
pYUB415_VapA-US-VapA	Expression of *vapA* in *R. equi* under the control of its own promoter	HygR		This study
pYUB415_VapA-US-VapB	Expression of *vapB* in *R. equi* under the control of the *vapA* promoter	HygR		This study
pYUB415_VapA-US-VapN	Expression of *vapN* in *R. equi* under the control of the *vapA* promoter	HygR		This study

Recombinant VapB was cloned into the (His_6_) tag vector pETite-N-His (Lucigen Expresso T7 cloning and expression system, catalog no. 49001-1). The gene was amplified from the *R. equi* PAM1593 virulence plasmid pVAPB (GenBank accession no. AM947676) using the primers pETite-N-His-VapB-FW and pETite-N-His-VapB-RV (Table 3). Cloning was performed according to the manufacturer’s instructions. The signal sequence in the pETite-N-His-VapB construct was deleted afterward using the primers pETite-N-His-VapB-SS-Deletion-FW and pETite-N-His-VapB-SS-Deletion-RV (Table 3). Cloning was performed according to an *in vivo* assembly cloning strategy published earlier (48). Therefore, the plasmid was amplified by inverted PCR with NEB Q5 high-fidelity DNA polymerase (NEB, catalog no. M0491) using the primers stated above, followed by template DNA digestion with DpnI (Thermo Fisher, catalog no. FD1703). For transformation, 2 μL of the PCR was mixed with 50 μL of E. coli NEB5α chemically competent cells (NEB, catalog no. C2987).

**TABLE 3:** PCR oligonucleotides used in this study

Oligonucleotide^*a*^	Sequence (5′–3′)^*b*^
Apr_FW	AGAAGTAAGCTTGATATCCATGTGCAGCTCCATCAG
Apr_RW	GCTATGGAATTCGATATCCTCATGAGCTCAGCCAATC
dfr_FW	CGGAATTCCATAGCAGGCTGAAGGTAGC
dfr_RV	GCAAACAAAGAGCTCCAGC
pETite-N-His-VapB-FW	CATCATCACCACCATCACATGATGAAGGCTCTTCATAAG*
pETite-N-His-VapB-RV	GTGGCGGCCGCTCTATTATTATGCAACCTCCCAGTTGTG*
pETite-N-His-VapB-SS-Deletion-FW	GTGATGGTGGTGATGATGCATATGT
pETite-N-His-VapB-SS-Deletion-RV	CACCACCATCACGCTGTGCTGGATTCCGGA†
pETite-N-His-VapN-FW	CATCATCACCACCATCACCAGCCGCTGGACGTTGGAG*
pETite-N-His-VapN-RV	GTGGCGGCCGCTCTATTACGCCCAGCTGCCCGTTC*
Seq_FW	CCCTATAGTGAGTCGTATTACATC
Seq_ RV	GTTCCCTTTAGTGAGGGTTAATAG
T7_FW	GCTCGCGATGTAATACGACTCACTATAGGG
T3_RV	TGAGTACTATTAACCCTCACTAAAGGGAAC
ufr_FW	CGGGTACCCGCAAAGGTCGGCTACC
ufr_RV	CCCAAGCTTACTTCTCCTTTCGGATGTCG
VapA_US_FW	CAGGCATGCAAGCTCAGGATATCCCAGGCGAAGACCTCAATCG*
VapA_RV	TAAAGGATCTTAATTAAGGATCCCTAGGCGTTGTGCCAGCTAC*
VapA_US_RV	CTTACTTCTCCTTTCGGACGTCG†
VapB_FW	CGACGTCCGAAAGGAGAAGTAAGATGATGAAGGCTCTTCATAAGACGG*
VapB_RV	TAAAGGATCTTAATTAAGGATCCTTATGCAACCTCCCAGTTGTGC*
VapB-GK_FW	GGGCCAGAAGGAAAGGTCTTCGATGGTGACGCG
VapB-GK_RV	TTCTGGCCCGTATACGTG
VapB A177G_FW	CCAGGCCGGTGGCGTTAGTGCTGTGGTGG
VapB A177G_RV	ACCGGCCTGGATATG
VapB A180T_FW	TGCAGTTAGTACCGTGGTGGGCGTTGG
VapB A180T_RV	ACTAACTGCACCGGCC
VapB L154V_FW	GTACAACGCCGTGGGGACTTACCTGAACAT
VapB L154V_RV	GGCGTTGTACTGAAAAG
VapB S137N_FW	TCTCTTCACAAATGACCTTCAGCGTCTC
VapB S137N_RV	TGTGAAGAGAGTCCC
VapB T156P_FW	CCCTCGGGCCGTACCTGAACATCAACTTCTTC
VapB T156P_RV	CCCGAGGGCGTTGT
VapN_FW	CGACGTCCGAAAGGAGAAGTAAGATGGGGAACGCGCGCAG‡
VapN_RV	TAAAGGATCTTAATTAAGGATCCCTACGCCCAGCTGCCCG‡

a FW, forward; RV, reverse.

b *, Underlined nucleotides indicate flanking sequences identical to the vector sequences flanking the cloning site; †, underlined nucleotides indicate an overlap homologous to the forward primer; ‡, underlined nucleotides, homologous region. For additional information, see the supplemental material.

Recombinant VapN was expressed from the hexahistidine tag vector pETite-N-His (Lucigen Expresso T7) cloning and expression system. The gene was amplified from the *R. equi* PAM1571 virulence plasmid pVAPN (GenBank accession no. KF439868) without its signal sequence using the primers pETite-N-His-VapN-F and pETite-N-His-VapN-R (Table 3). Cloning was performed according to the manufacturer’s instructions. For all E. coli-expressed Vaps, the resulting proteins had no signal sequence, which would, in *R. equi*, be required to export the Vap but which do not exist in the secreted form of the protein.

Mutant forms of recombinant VapB were generated by site-directed mutagenesis. Therefore, inverted primer pairs (Table 1) with homologues overlaps were synthesized (Thermo Fisher Scientific), with one primer containing the substituted nucleotides. Then, the whole plasmid pETite-N-His-VapB (the one without the secretion sequence) was amplified by inverted PCR and assembled *in vivo* as described above. The VapA mutant plasmid pET28(+)-VapA-DM was synthesized by BioCat GmbH (Heidelberg, Germany).

For *vapA*, *vapB*, and *vapN* with *vapA* upstream sequences, VapA with its 857 upstream nucleotides (“VapA-US”) directing its expression in *R. equi* was amplified from the *R. equi* 103 pVAPA plasmid using the primer pair VapA_US_FW and VapA_RV. These 857 bp are composed of the 545-bp upstream region of *vapA* as in Jain et al. (22) and an extra 312 bp upstream. The same general construct containing *vapB* with the *vapA* upstream region for gene regulation (sequence in Fig. S1 in the supplemental material) was obtained by amplification of the *vapA* upstream region from pVAPA using the primers VapA_US_FW and VapA_US_RV and by amplification of the *vapB* gene from the *R. equi* PAM1593 pVAPB virulence plasmid with the primers VapB_FW and VapB_RV. The corresponding *vapN* construct with upstream *vapA* expression-mediating sequences was obtained by amplification of the *vapA* upstream region, as stated above, and amplification of the *vapN* gene from *R. equi* PAM1571 pVAPN virulence plasmid using the primers VapN_FW and VapN_RV. For each construct, the gene and the *vapA* upstream region were cloned into EcoRV/BamHI-digested pYUB415 shuttle vector (obtained from William Jacobs, Albert Einstein College of Medicine, Bronx, NY) by sequence- and ligase-independent cloning (SLIC) (49). To this end, 2 μg of pYUB415 vector DNA was digested with EcoRV and BamHI and separated by agarose gel electrophoresis, followed by gel extraction of the excised band using a PCR cleanup kit (Macherey & Nagel, catalog no. 740609.50). The *vap* gene and *vapA* upstream fragments were amplified by PCR using *Pfu* DNA polymerase. The remaining DNA template was removed by DpnI digestion, and the PCR products were purified using a PCR cleanup kit. Then, 1 μg of vector and 1 μg of insert DNA were treated with 0.5 U of T4 DNA polymerase to generate single-stranded DNA overhangs (T4 DNA polymerase shows exonuclease function in the absence of deoxynucleoside triphosphates). The annealing reaction was set up by mixing 450 ng of vector with equimolar amounts of insert, 1× T4 DNA ligase buffer and water. The mixture was incubated for 30 min at 37°C, and 5 μL was immediately used for transformation into chemically competent E. coli TG1. The cells were recovered for 1 h at 37°C and plated onto LB selection plates containing 100 μg/mL ampicillin.

*vapB* was deleted as follows. Two regions were amplified from the PAM1593 virulence plasmid upstream (upstream flanking region [ufr]) and downstream (downstream flanking region [dfr]) of the *vapB* gene. The ufr (368 bp) forward (FW) and reverse (RV) primers were flanked by KpnI and HindIII sites, respectively. The dfr (382 bp) FW primer was flanked by EcoRI, while the RV primer included an endogenous SacI restriction site. pBluescript was digested with KpnI and HindII and ligated with ufr digested using the same enzymes. pBluescript-ufr was then digested with EcoRI and SacI and ligated with dfr digested using the same restriction enzymes, resulting in pBluescript-ufr-dfr. The plasmid was cut between the flanking sites with EcoRV and combined with the apramycin resistance cassette *aacC*4 amplified from pVK173T with the primers Apr_FW and Apr_RV containing homologous overhangs using sequence and ligase independent cloning (50). The resulting insert, ufr-aacC4-dfr, was amplified by PCR using the primers T7 and T3, and the amplicon was digested with NruI and ScaI. The suicide plasmid pAPvlacZ (51) was digested using the same enzymes, and the larger of the two resulting fragments (harboring the *vapA* promoter upstream of the β-galactosidase gene but no *R. equi*-compatible origin of replication) was ligated with the digested PCR fragment, resulting in the pAPvlacZ-ufr-aacC4-dfr suicide plasmid. The final plasmid was electroporated into PAM1593, and transformants were selected for apramycin resistance. Blue apramycin-resistant colonies were picked and cultivated in apramycin-containing media. White colonies on selective agar plates (double-crossover events) lacked the suicide plasmid backbone and *vapB* open reading frame. Mutants were confirmed by sequencing (Seq_FW and Seq_RV).

### Gene expression.

Recombinant hexahistidine-tagged Vaps, as described above, and recombinant VapA, as described previously (8), were expressed in E. coli BL21(DE3)pLysS (NEB, catalog no. C2527H) and purified as described previously (8). Hexahistidine-tagged listeriolysin O (LLO) was kindly provided by Daniel A. Portnoy (University of Berkeley) (52). Rhodococcus equi was transformed based on the protocol by (53). Electrocompetent *R. equi* were thawed on ice and pipetted into a precooled electroporation cuvette (2 mm; Biozym, catalog no. 748020). Bacteria were mixed with 1 to 2 μg of plasmid DNA and incubated on ice for 5 min. The cuvette was placed in the micropulser (Bio-Rad) holder, and the solution was pulsed at 2.5 kV (program EC2). Bacteria were immediately mixed with 1 mL of BHI medium (Becton Dickinson) and left on ice for 1 min. The culture was transferred into culture tubes, incubated at 30°C and 200 rpm for 1 h, plated onto BHI agar containing 100 μg/mL apramycin and 100 μg/mL hygromycin B, and cultivated at 30°C for 2 days.

### Triton X-114 partitioning experiments.

The protocol used for the TX-114 partitioning experiments was adapted a previously described method (54). For precondensation of TX-114 (Merck Sigma-Aldrich, catalog no. 93421), 1 mL of (TX-114) was mixed with 49 mL of deionized water, followed by incubation on ice until clear. Phase separation was performed overnight at 37°C. The upper aqueous phase was discarded and replaced by TBS (20 mM Tris, 150 mM NaCl [pH 7.4]), and the step was repeated twice. The lower (detergent) phase was further used as TX-114. Next, 100 μg of the indicated protein (Roti-Quant protein assay; Carl Roth, Karlsruhe, Germany) was mixed with TX-114, 10× TBS, and Milli-Q water to obtain a 2% TX-114/TBS solution. The mixture was cleared on ice and incubated for 10 min at 37°C. For complete phase separation, the solution was centrifuged for 5 min at 300 × *g* at 25°C. The upper aqueous phase was taken and mixed with TX-114 to produce a 2% solution. The lower detergent phase was mixed with an equal volume 0.1% TX-114/TBS. Both solutions were cleared on ice, and the phases were separated by warming and centrifugation. This washing step was repeated twice, the aqueous and detergent phases were separately combined in Pyrex tubes, and the contained proteins were precipitated overnight with 4 volumes of acetone at –20°C. The precipitates were collected by centrifugation at 3,000 × *g* for 10 min at 4°C, and the pellet was dried under nitrogen gas. Samples were analyzed by SDS-PAGE and staining with colloidal Coomassie G-250 (55). All polyacrylamide gels in the study were 12% acrylamide, and the molecular mass standards were from Thermo Fisher Scientific (PagRuler prestained protein ladder, catalog no. 26616). Hen egg lysozyme was obtained from Merck Sigma-Aldrich (catalog no. 62970), and carbonic anhydrase was obtained from Serva (catalog no. 15882).

### Microscopy.

Preparation of (infected) macrophage samples and their analyses were as described previously (8). For the LT (LysoTracker Red DND-99; Thermo Fisher Scientific, catalog no. 7528) analysis, a positive colocalization was defined as any fluorescence signal above background. For phagosome membrane markers, positive colocalization was defined as most of the phagosome membrane having an above-background signal either encircling the bacteria or covering them. The murine monoclonal antibody to VapA was obtained from Santa Cruz Biotechnology (catalog no. sc-390576).

Determination of lysosome size was done as described previously (8), except that 100 μg/mL rVap was used and that the samples were counterstained with an antibody to lysosome-associated membrane protein-1 (LAMP-1; clone 1D4B; Santa Cruz Biotechnology, catalog no. sc-19992). A Carl Zeiss Laser Scanning Microscope LSM510 in expert modus, and Fiji software was used for size quantification.

### Liposome production.

The lipids used in the present study are listed in Table 4. The lipids were dissolved in chloroform, flushed with liquid nitrogen, and kept in glass flasks at –20°C. For each experiment, 400 nmol of the respective lipid mixture or, in case of complex lipid mixtures such as total brain lipids, 500 μg of the lipid mixture was dried under nitrogen gas in a 2-mL round-bottom plastic tube and rehydrated in 25 μL of hydration buffer (as indicated below) for 1 h in a 60°C water bath. An Avanti Mini Extruder (Sigma-Aldrich, catalog no. 610020-1EA) was assembled. The turbid lipid mixture was ultrasonicated for 90 s (Branson Ultrasonic CL-40549) and filled into an extruder-compatible 1-mL Hamilton syringe (Sigma-Aldrich, catalog no. 610017-1EA). Whatman Nuclepore Track-Etched polycarbonate membranes (pore size, 0.2 μm) were inserted. The lipid mixture was equilibrated on the Mini Extruder heating bloc (Sigma-Aldrich, catalog no. 610024) for 10 min at 60°C and then pressed 21 times through the extruder. The resulting liposomes were stored at 4°C until use, with a maximum of 5 days.

**TABLE 4:** Lipids and lipid mixtures used in this study

Lipid (mixture)	Origin	Source, catalog no.	Stock concn (mg/mL) in chloroform
Asolectin	Soy bean	Sigma, 11145	10
Cholesterol	Sheep	Avanti, 700000P	10
Phosphatidate	Chicken egg	Avanti, 840101	10
Phosphatidylcholine^*a*^	Synthetic	Avanti, 850375	10
Phosphatidylethanolamine^*b*^	Synthetic	Avanti, 850725	10
Phosphatidylserine	Pig	Avanti, 840032	10
Sphingomyelin	Pig	Avanti, 860062	25
Total brain extract	Pig	Avanti, 131101	10

a DOPC, 18:1 (Δ9-cis).

b DOPE, 18:1 (Δ9-cis.).

### Liposome flotation assay.

The binding of proteins to liposomes was investigated in a flotation assay, as previously described (56). Liposomes were hydrated in 50 mM MES/150 mM NaCl-Puffer (pH 4.5) and mixed into a 50-μL volume for 400 nmol lipid that was mixed with 50 μL of protein solution (100 μg/mL), followed by incubation at 37°C for 1 h. Then, 100 μL of 60% sucrose (771.9 g/L in MES/NaCl buffer; pH 4.5) was added, carefully mixed, placed in Beckman Ultra-Clear tubes (Beckman, catalog no. 344090), and overlaid with 250 μL of 25% sucrose solution (275.9 g/L in MES/NaCl buffer; pH 4.5), followed by 50 μL of MES/NaCl buffer (50 mM MES/NaCl 150 mM; pH 4.5). Centrifugation in a Beckman MLS-50 rotor was performed for 1 h at 50,000 rpm at 20°C (Accel/Decel 5). The lower and middle phases were carefully removed with Hamilton syringes in a volume of 200 μL each. One-fifth of the remaining upper phases was analyzed by SDS-PAGE and, for comparison, one-fifth of the added protein corresponding to 1 μg was also analyzed. In negative controls, liposomes were replaced by MES/NaCl buffer (pH 4.5). Before addition to liposomes, protein solutions were thawed on ice and centrifuged at 13,000 rpm and 4°C for 10 min in a tabletop microcentrifuge. The supernatant was removed, and its protein content analyzed by Bradford assay (Roti-Quant; Carl Roth) using BSA as a standard. Protein aliquots were used only once.

Liposome samples were run on 15% SDS-polyacrylamide gels and stained with colloidal Coomassie blue according to the study by Dyballa and Metzger (55). Protein signal intensities were quantified using software Fiji (57). The signal strength was compared to the signal obtained with the total protein amount placed in the binding assay.

### Liposome permeabilization assay.

Liposomes were generated above but with phosphate-buffered saline (PBS [pH 7.0]), containing 100 mM 5(6)-carboxyfluorescein (CF; Sigma-Aldrich, catalog no. 21877) to incorporate a self-quenching concentration of the fluor. Unincorporated CF was removed by gel filtration using Sephadex-G25 Fine (GE Healthcare) with gravity flow. Work was done in a dimly lit room to not permanently inactivate fluorescence. Eventually, 400 nmol of starting lipid was contained as liposomes in 100 μL of PBS (pH 7.0). No acidic buffer was used because the fluorescence of leaking CF would have been quenched immediately. Liposomes were filled in one well of a flat-bottom 96-well plate in a 37°C heated BioTek Instruments FLx800 fluorescence reader. Fluorescence was recorded at λ_ex/em_ 485/516 nm, 100 μL of protein solution (50 μg/mL) was added per well, and emission was recorded every 60 s for 60 min. To determine the maximal possible emission, all samples received a final concentration of 0.2% TX-100 (Carl Roth, catalog no. 3051.2). In negative-control samples, PBS was added instead of liposomes. In all samples, fluorescence was detected for 1 min without adding protein to calibrate the system; protein was then added, and all samples received a final concentration of 0.2% TX-100 after 1 h to quantify maximum fluorescence signals.

### Proteolysis/DQ-BSA degradation.

RAW 264.7 cells were seeded into 24-well plates and allowed to settle overnight. Cells were incubated for 20 h (i) with 50 μg/mL rVapA, rVapB, or rVapN; with (ii) 40 nM BafA1 dissolved in medium; or (iii) left untreated. After 20 h, the cells were incubated with 10 μg/mL DQ-BSA (Thermo Fisher Scientific, catalog no. D12051) in full DMEM and the respective protein or drug for 2 h. Cells were washed using PBS and incubated for 2 h with medium containing BafA1 or recombinant protein.

### Flow cytometry.

Infected macrophages were prepared as for the microscopic experiments. For the gating strategy in LT experiments, the threshold for event counting was set using forward scatter height (FSC-H), and only events of >200,000 U were measured. Cells were gated for viability, plotting the forward scatter area (FSC-A) against the side scatter area (SSC-A). Outliers were excluded, and harsh gating was unnecessary due to the exclusion of debris using the threshold. Only cells inside the viability gate were plotted: FSC-A against FSC-H. Cells with a large area or height were excluded in a gate to discriminate against doublets. A nesting gate was applied to the filter detecting light from LT (590 nm; FL-2 585/40), excluding debris, dead cells, and doublets. The target population was now gated using a horizontal gate, allowing an error margin of 10% in untreated samples. The same horizontal gate was then applied to every subsequent sample. Gating in DQ-BSA degradation experiments was performed using the same gates and thresholds. A nesting gate was applied for DQ-Red (590 nm; FL-2 585/40). A BD Accuri C6 cytometer was used for these analyses.

### Vap structure prediction and comparison.

Vap structures predicted using AlphaFold2 (https://www.nature.com/articles/s41586-021-03819-2) were downloaded from the AlphaFold Protein Structure Database (https://academic.oup.com/nar/advance-article/doi/10.1093/nar/gkab1061/6430488). The structural overlay and the figure were prepared using PyMOL.
