# Supplementary material for: Differential Effects of Rhodococcus equi Virulence-Associated Proteins on Macrophages and Artificial Lipid Membranes
Source: Microbiol Spectr. 2023 Feb 14;11(2):e03417-22. doi: 10.1128/spectrum.03417-22 (PMC10100859; doi:10.1128/spectrum.03417-22)
Supplement: Supplemental file 1 — Fig. S1. Download spectrum.03417-22-s0001.pdf, PDF file, 0.4 MB [file spectrum.03417-22-s0001.pdf]

Supplemental Figure 1.

Sequence of the codon-optimized gene for the *Escherichia coli* orthologue of VapA. The synthetic gene (NCBI Reference Sequence of the protein: WP_001304589.1) was used for construction of the plasmid as stated in the *Materials and Methods* section. Restriction endonuclease recognition sites and nucleotide numbers are indicated above the sequence.

```
1                                      SacI    XhoI
BstEII
CGAATTGGCGGAAGGCCGTCAAGGCCACGTGTCTTGTCCAGAGCTCCTCGAGGTCATGGT
---------+---------+---------+---------+---------+---------+
GCTTAACCGCCTTCCGGCAGTTCCGGTGCACAGAACAGGTCTCGAGGAGCTCCAGTACCA
                                                       M__V_

61
TACCACCATGACCAAATGTAGCAATCTGCGTCAGCAGATTATGGATGATGTTCAGCGTCG
---------+---------+---------+---------+---------+---------+
ATGGTGGTACTGGTTTACATCGTTAGACGCAGTCGTCTAATACCTACTACAAGTCGCAGC
_T__T__M__T__K__C__S__N__L__R__Q__Q__I__M__D__D__V__Q__R__R_

121
TTATGGTGAGTATCTGGATAAAGATAAAGTGAGCTGCATCACCAGCAAAATTGCAGCAGC
---------+---------+---------+---------+---------+---------+
AATACCACTCATAGACCTATTTCTATTTCACTCGACGTAGTGGTCGTTTTAACGTCGTCG
_Y__G__E__Y__L__D__K__D__K__V__S__C__I__T__S__K__I__A__A__A_

181
AGAAAACAAATATCCGGCAAAAACCACCCTGGCAAGCGCCATTTTTTACATTAAAGTTGA
---------+---------+---------+---------+---------+---------+
TCTTTTGTTTATAGGCCGTTTTTGGTGGGACCGTTCGCGGTAAAAAATGTAATTTCAACT
_E__N__K__Y__P__A__K__T__T__L__A__S__A__I__F__Y__I__K__V__D_

241
TACCCAGATTACCAGCGAAGGTGGTAAACATTTTAGCGGTAATGCCGGTGGTCTGAGCAG
---------+---------+---------+---------+---------+---------+
ATGGGTCTAATGGTCGCTTCCACCATTTGTAAAATCGCCATTACGGCCACCAGACTCGTC
_T__Q__I__T__S__E__G__G__K__H__F__S__G__N__A__G__G__L__S__S_

301
TCCGGGTGGTGGTGTTCTGTTTGGTGATCTGTATACCGATGATCTGGATGACCTGTATAC
---------+---------+---------+---------+---------+---------+
AGGCCCACCACCACAAGACAAACCACTAGACATATGGCTACTAGACCTACTGGACATATG
_P__G__G__G__V__L__F__G__D__L__Y__T__D__D__L__D__D__L__Y__T_
```

```
361                          AgeI
CAATACCGTTAGCTTTCAGATTACAATGACACCGGTTTTTTGCAGCGTGCTGTTTTTTGA
---------+---------+---------+---------+---------+---------
+GTTATGGCAATCGAAAGTCTAATGTTACTGTGGCCAAAAAACGTCGCACGACAAAAAACT
_N__T__V__S__F__Q__I__T__M__T__P__V__F__C__S__V__L__F__F__D_

421
TAGCGCCTCAAATCTGCTGGGTCATTTTGAAGGTGGTGGCGTTAGCACCGTTAGCGGTGT
---------+---------+---------+---------+---------+---------+
ATCGCGGAGTTTAGACGACCCAGTAAAACTTCCACCACCGCAATCGTGGCAATCGCCACA
_S__A__S__N__L__L__G__H__F__E__G__G__G__V__S__T__V__S__G__V_

481      AgeI                      NdeI     KpnI
TGCGGGTGGCACCGGTAGCTGGTCATAAACGCATATGGGTACCTGGAGCACAAGACTGGC
---------+---------+---------+---------+---------+---------
ACGCCCACCGTGGCCATCGACCAGTATTTGCGTATACCCATGGACCTCGTGTTCTGACCG
_A__G__G__T__G__S__W__S__*__

541
CTCATGGGCCTTCCGCTCACTGC
---------+---------+---
GAGTACCCGGAAGGCGAGTGACG
```
